# Chocolate Consumption and Risk of Coronary Heart Disease, Stroke, and Diabetes: A Meta-Analysis of Prospective Studies

**DOI:** 10.3390/nu9070688

**Published:** 2017-07-02

**Authors:** Sheng Yuan, Xia Li, Yalei Jin, Jinping Lu

**Affiliations:** Department of Geratology, Zhongnan Hospital of Wuhan University, Wuhan University, Wuhan 430071, China; whumedor@163.com (S.Y.); mengxingshunking@163.com (X.L.); hhl201802@163.com (Y.J.)

**Keywords:** chocolate consumption, coronary heart disease, stroke, diabetes, meta-analysis

## Abstract

Although epidemiological studies have examined the role of chocolate in preventing cardiometabolic disease, the results remain inconsistent. Herein, we conducted a meta-analysis of prospective studies to determine the association between chocolate intake and risk of coronary heart disease (CHD), stroke, and diabetes. A systematical search in PubMed and Embase through March 2017, together with reference scrutiny of relevant literatures, was performed to identify eligible studies. Relative risks (RRs) and 95% confidence intervals (CIs) were pooled using random effect models. Fourteen prospective studies of primary prevention with 508,705 participants were finally included, with follow-up durations ranging from 5 to 16 years. The summary RRs for the highest versus lowest chocolate consumption were 0.90 (95% CI: 0.82–0.97; *n* = 6) for CHD, 0.84 (95% CI: 0.78–0.90; *n* = 7) for stroke, and 0.82 (95% CI: 0.70–0.96; *n* = 5) for diabetes. Dose–response meta-analysis suggested a nonlinear association of chocolate consumption with all outcomes. For both CHD and stroke, there was little additional risk reduction when consuming chocolate ≥3 servings/week (one serving was defined as 30 g of chocolate). For diabetes, the peak protective effect of chocolate emerged at 2 servings/week (RR: 0.75, 95% CI: 0.63–0.89), with no benefit observed when increasing consumption above 6 servings/week. In conclusion, chocolate intake is associated with decreased risks of CHD, stroke, and diabetes. Consuming chocolate in moderation (≤6 servings/week) may be optimal for preventing these disorders.

## 1. Introduction

Cardiovascular disease, including coronary heart disease (CHD) and stroke, remains the leading cause of morbidity and mortality worldwide [1]. It was estimated that nearly 16.7 million deaths in 2010 were attributable to these diseases, with projections suggesting a staggering 23.3 million by 2030 [2]. Diabetes is also a widespread chronic disease with severe clinical sequelae, such as cardiovascular disease, renal dysfunction, retinopathy, and diabetic cardiomyopathy. The global prevalence of diabetes is rising progressively and is estimated to increase from 366 million cases in the year 2011 to 552 million cases in 2030 [3]. Therefore, effective prevention strategies for these cardiometabolic disorders are essential to improve public health and to relieve the social and economic burdens.

Diet is one of the key lifestyle elements involved in the prevention and control of cardiometabolic disease. Chocolate is a highly popular dietary food throughout the world, and has gained increasing attention for its potential benefits in cardiometabolic health. A number of experimental and clinical studies have indicated a protective role of chocolate against oxidative stress, inflammation, endothelial dysfunction, and atherogenesis [4]. These salutary effects have been confirmed in recent meta-analyses of feeding trials, supporting the favorable impact of chocolate on cardiometabolic risk factors such as blood pressure, lipid profiles, flow-mediated dilatation, and insulin sensitivity [5,6,7]. Generally, the evidence so far regarding chocolate consumption and improved vascular function is strong, but the evidence showing reduced risk of cardiovascular disease is weaker. Moreover, there are limited studies centering on the association of chocolate intake with hard cardiometabolic outcomes (e.g., CHD, stoke, and diabetes), and the overall results remain inconclusive. We therefore conducted a meta-analysis of pertinent prospective studies to comprehensively evaluate the role of chocolate in prevention of CHD, stoke, and diabetes.

## 2. Materials and Methods 

### 2.1. Search Strategy

This meta-analysis was performed in accordance with the meta-analysis of observational studies in epidemiology guideline provided in [8]. We conducted an electronic literature search in PubMed and Embase databases until March 2017 to identify eligible publications. The search terms included “chocolate” or “cocoa”. We combined these terms with free-text word searches that included “coronary heart disease”, “coronary artery disease”, “ischemic heart disease”, “stroke”, “cardiovascular disease”, and “diabetes”. In addition, the reference lists of retrieved articles and reviews were manually checked to include additional studies. 

### 2.2. Inclusion Criteria

To be included, the studies should meet all of the following requirements: (1) the studies were cohort or nested case–control studies; (2) the exposure of interest was chocolate consumption, defined as consumption of any type of chocolate products (e.g., dark, white, or milk chocolate); (3) the outcomes of interest included CHD, stroke, or diabetes; (4) the risk estimates of outcomes (e.g., relative risks, RRs) were provided. For studies included in the dose–response meta-analysis, the risk estimates must be reported for at least three categories of chocolate intake, and for each category, the number of cases and participants or person-years were also provided (or with details available to calculate them). Reviews, abstracts, and duplicates were excluded. The study selection process was performed by two independent reviewers (S.Y. and X.L.) according to the inclusion and exclusion criteria, with disagreements settled by discussion with a third reviewer (Y.J.). 

### 2.3. Data Collection and Quality Evaluation

The main characteristics of studies were abstracted, including study author, publication year, population origin, sample size, age at inclusion, ascertainments of exposure and outcome, country, duration of follow-up, outcomes measured, adjustments in multivariate models, and the maximally adjusted risk estimates. The methodological quality of included studies was assessed according to the Newcastle–Ottawa Scale (NOS) [9], with the following three major aspects: selection of participants (score 0–4), comparability between study groups (score 0–2), and ascertainment of outcomes (score 0–3). A study was considered of high quality if it had an NOS score of ≥7. All data extraction and quality assessment were performed by two independent reviewers (S.Y. and X.L.), with discordance resolved by consulting with a third reviewer (Y.J.). 

### 2.4. Statistical Analyses

In this study, the summary risk estimates were presented as RRs with corresponding 95% confidence intervals (CIs). We pooled the study-specific risk estimates of CHD, stroke, and diabetes for the highest versus lowest categories of chocolate consumption using random effect models. Heterogeneity among the included studies was detected by the Cochrane Q test, with *p* values of <0.1 indicating significance. We also reported the heterogeneity as low, moderate-to-high, and substantial with *I*^2^ values of <25%, 25–75%, and >75%, respectively [10]. Subgroups analyses (if available) were performed according to gender and follow-up duration, with difference between subsets confirmed by the Altman and Bland test [11]. Leave-one-out sensitivity analysis was conducted to assess the robustness of pooled results. Publication bias was explored by Egger’s test. If publication bias was found, we used the “trim and fill” strategy to adjust the bias and then re-computed the results [12].

To evaluate the dose–response relationship between chocolate consumption and risk of each outcome, we firstly standardized the chocolate intake levels across studies using a common measure (i.e., servings/week), and one serving of chocolate was assumed to approximate 30 g of chocolate [13]. The mean or median level of chocolate intake for each category was then assigned to each corresponding RR. If the mean or median level was not provided, we assigned the midpoint of the upper and lower boundaries of each category as the average consumption level. When the upper boundary of the highest intake category was not available, we estimated the midpoint to be 1.5-times the lower boundary. When the lowest category was open-ended, we assumed its lower boundary as zero. 

We performed a two-stage random-effects dose–response meta-analysis to examine the potential nonlinear association between chocolate consumption and risk of CHD, stroke, and diabetes. In the first stage, a restricted cubic spline model with three knots at the 10th, 50th, and 90th percentiles of chocolate intake was estimated using generalized least-square regression considering the correlation within each set of published RRs, as proposed by Orsini et al. [14]. In the second stage, the restricted maximum likelihood method in a random-effects meta-analysis was used to pool the study-specific RRs [15]. A *p*-value for nonlinearity was calculated by testing the null hypothesis that the coefficient of the second spline is equal to zero. 

All statistical analyses were performed using STATA 13.0 (StataCorp, College Station, TX, USA) and R 3.2.5 (The R Foundation for Statistical Computing, Vienna, Austria) software, and a two-sided *p*-value of <0.05 was considered as significant. 

## 3. Results

### 3.1. Study Search

**Figure 1:**
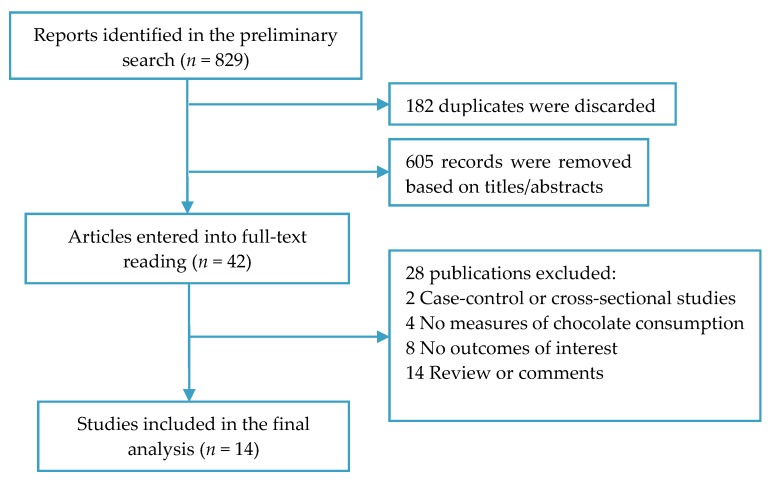
Flow diagram of study selection.

A total of 829 publications were preliminarily identified, of which 182 duplicates and 605 irrelevant articles were discarded. After full-text review of the remaining 42 records, 28 studies were excluded because of failure to meet the eligibility criteria. As a consequence, 14 prospective studies [13,16,17,18,19,20,21,22,23,24,25,26,27,28] were included in the present meta-analysis (Figure 1). 

### 3.2. Characteristics of Studies

The main characteristics of the selected studies are summarized in Table 1. These prospective studies were published from 2007 through 2017, with the follow-up durations ranging from 5 to 16 years. Five studies were conducted in the United States, four in Sweden, two in Japan, and one each in UK, Germany, and Australia. In total, we identified 144,822 participants and 7267 cases for CHD, 231,038 participants and 8197 cases for stroke, and 132,845 participants and 13,271 cases for diabetes. All participants were free from CHD, stroke, or diabetes at inclusion, with age varying from 35 to 84 years. Chocolate consumption was ascertained using a validated food frequency questionnaire in all studies except one, and the outcome events were frequently verified through International Classification of Disease (ICD) codes. The majority of chocolate consumed in the included studies was milk or dark chocolate. Among the studies, the most commonly adjusted confounders were age, smoking status, alcohol consumption, dietary energy, and physical activity. The study quality scores varied from 7 to 9, and the mean NOS score was 8.1, suggesting the presence of high methodological quality.

### 3.3. Chocolate Consumption and Risk of CHD

**Figure 2:**
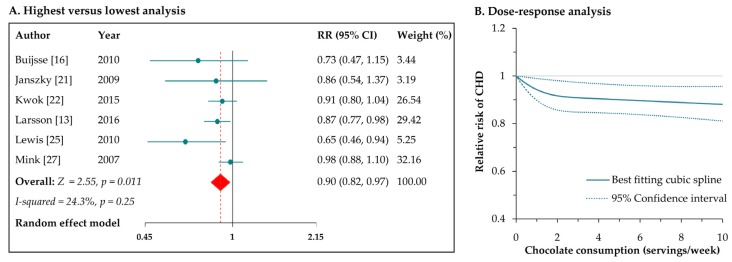
Meta-analyses of chocolate consumption and risk of coronary heart disease (CHD). CI: confidence interval; RR: relative risk.

Six prospective studies [13,16,21,22,25,27] reported the risk of CHD associated with chocolate consumption. The pooled RR of CHD for the highest versus lowest intake of chocolate was 0.90 (95% CI: 0.82–0.97; Figure 2A), with low heterogeneity across studies (*I*^2^ = 24.3%, *p* = 0.25). Regarding CHD subtype, the RR was 0.86 (95% CI: 0.77–0.96) for myocardial infarction. The results were consistent for studies with follow-up duration of <10 years (RR: 0.72, 95% CI: 0.57–0.92) and for studies with follow-up duration of ≥10 years (RR: 0.92, 95% CI: 0.86–0.99). Leave-one-out sensitivity analysis had no significant influence on the pooled results. There was no indication of publication bias from Egger’s test (*p* = 0.12).

In the dose–response meta-analysis of five studies [13,16,21,22,25], we found a curvilinear association between chocolate consumption and risk of CHD (*p* for nonlinearity = 0.006; Figure 2B). Compared with no intake, the RRs (95% CIs) of CHD across chocolate consumption levels were 0.94 (0.90–0.99), 0.91 (0.85–0.97), 0.89 (0.83–0.95), and 0.88 (0.81–0.95) for 1, 3, 7, 10 servings/week, respectively. In general, there appeared to be little additional reduction in risk when consuming chocolate ≥ 3 servings/week.

### 3.4. Chocolate Consumption and Risk of Stroke

**Figure 3:**
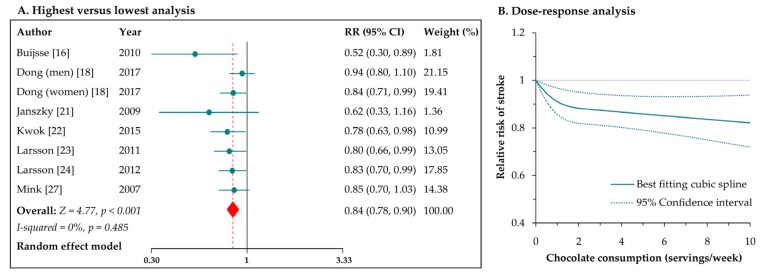
Meta-analyses of chocolate consumption and risk of stroke.

Eight reports from seven studies [16,18,21,22,23,24,27] estimated the risk of stroke for the highest versus lowest level of chocolate consumption; the pooled results indicated a significant inverse association (RR: 0.84, 95% CI: 0.78–0.90; Figure 3A), with no evidence of heterogeneity (*I*^2^ = 0%, *p* = 0.49). With regard to stroke subtypes, the RRs were 0.87 (95% CI: 0.78–0.96) for cerebral infarction and 0.83 (95% CI: 0.71–0.97) for hemorrhagic stroke. In the stratified analysis by gender, the RRs of total stroke were 0.87 (95% CI: 0.79–0.97) and 0.84 (95% CI: 0.74–0.94), respectively, for male and female participants (*p* for interaction = 0.66). Besides, the results were consistent for studies with follow-up durations of <10 years (RR: 0.56, 95% CI: 0.37–0.85) and for studies with follow-up duration of ≥10 years (RR: 0.85, 95% CI: 0.79–0.91). The pooled risk of total stroke was not obviously modified in the sensitivity analysis by excluding one study at a time. Egger’s test suggested the presence of publication bias (*p* = 0.008). After introducing the “trim and fill” method to adjust this bias, the overall risk estimate remained significant in favor of chocolate intake (RR: 0.86, 95% CI: 0.79–0.92).

Seven reports from six studies [16,18,21,22,23,24] were included in the dose–response meta-analysis. There was a nonlinear correlation between chocolate intake and risk of stroke (*p* for nonlinearity = 0.001; Figure 3B). The RRs (95% CIs) of stroke were 0.91 (0.86–0.97) for 1 serving/week, 0.87 (0.81–0.94) for 3 servings/week, 0.85 (0.76–0.93) for 7 servings/week, and 0.83 (0.72–0.94) for 10 servings/week. Generally, there seemed to be little further reduction in stroke risk when increasing chocolate consumption above 3 servings/week.

### 3.5. Chocolate Consumption and Risk of Diabetes

**Figure 4:**
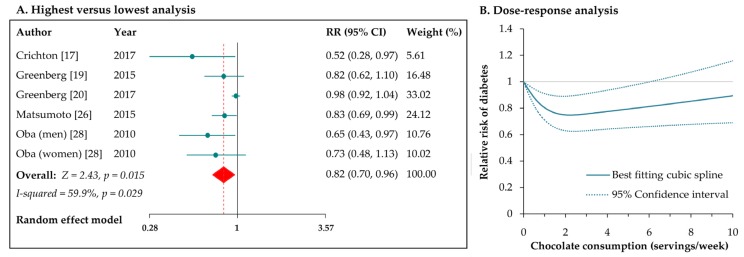
Meta-analyses of chocolate consumption and risk of diabetes.

Six independent reports from five studies [17,19,20,26,28] estimated the risk of diabetes for chocolate consumption. Compared with the lowest intake, the highest intake of chocolate was associate with a reduced risk of developing diabetes (RR: 0.82, 95% CI: 0.70–0.96; Figure 4A), with moderate heterogeneity among the studies (*I*^2^ = 60%, *p* = 0.03). After excluding the study by Greenberg et al. [19], the heterogeneity was remarkably reduced (*I*^2^ = 0%, *p* = 0.55), with the pooled result of the remaining studies maintaining significance (RR: 0.78, 95% CI: 0.68–0.89). In the stratified analysis by sex, the RRs were 0.79 (95% CI: 0.65–0.96) for men and 0.92 (95% CI: 0.72–1.17) for women (*p* for interaction = 0.34). Similarly, the risks of diabetes were not different between subsets of studies with follow-up durations of below or over 10 years (*p* for interaction = 0.51). Exclusion of single studies in sequence did not alter the inverse association between chocolate intake and risk of diabetes. There was an indication of publication bias from Egger’s test (*p* = 0.001); the summary risk estimate was marginally significant after the use of “trim and fill” method (RR: 0.92, 95% CI: 0.78–1.08).

In dose–response meta-analysis of the six reports, we observed a curvilinear association between chocolate intake and risk of diabetes (*p* for nonlinearity < 0.001; Figure 4B). The RRs (95% CIs) of diabetes were 0.80 (0.71–0.91), 0.76 (0.63–0.91), 0.83 (0.67–1.03), and 0.89 (0.69–1.16), respectively, for chocolate consumptions of 1, 3, 7, 10 servings/week. In general, the dose–response pattern was J-shaped, and the peak reduction in risk occurred at an intake of 2 servings/week (RR: 0.75, 95% CI: 0.63–0.89), with no protective effects observed when consuming chocolate > 6 servings/week. 

## 4. Discussion

Clinical researches on the health effects of chocolate have accelerated in recent years, in particular on cardiometabolic health. The present work of 14 prospective cohort studies, with 508,705 participants from six countries and 7267 CHD cases, 8197 stroke cases, and 13,271 diabetes cases provides the most robust and reliable evidence to date of how chocolate consumption may affect risk of cardiometabolic diseases. Compared with the lowest intake, the highest consumption of chocolate was associated with decreased risks of CHD, stroke, and diabetes. Dose–response meta-analyses suggested nonlinear associations of chocolate intake with all these disorders. There seemed little further reduction in risks of CHD and stroke when increasing the intake of chocolate over 3 servings/week; for diabetes, consuming 2 servings/week of chocolate had the greatest protective effect, with no benefits for an intake of >6 servings/week.

In line with our findings, previous meta-analyses also indicated that higher chocolate intake was related to lower risks of CHD and stroke. In the study of Kwok et al. [22], the highest chocolate consumption conferred a 29% and 21% risk reduction in incident CHD and stroke, respectively, in comparison to the lowest consumption. Similarly, Larsson et al. pooled the results of five prospective studies and found that the highest versus lowest category of chocolate consumption was associated with a decreased risk of developing stroke (RR: 0.81, 95% CI: 0.73–0.90) [13]. However, categories of chocolate intake differed between the studies included, which might complicate the interpretation for the combined results. In this respect, a dose–response meta-analysis with restricted cubic spline functions offers a solution to the problem [29], but no previous meta-analysis has been performed to explore the dose–response pattern of the relationship between chocolate consumption and risks of CHD and stroke. Moreover, our study is the first meta-analysis investigating the protective role of chocolate consumption against diabetes.

The cardiometabolic benefits of chocolate intake are biologically plausible. Chocolate is an abundant source of flavanols such as epicatechin, catechin, and procyanidins [30]. The flavanols may offer cardiometabolic protection through several mechanisms, including antihypertensive, antiplatelet, antioxidant, and anti-inflammatory effects [31]. A number of studies have examined the effects of cocoa or chocolate products on cardiovascular risk factors. One recent meta-analysis summarized the results of 35 feeding trials, and suggested that flavanol-rich chocolate and cocoa products caused a significant reduction in both systolic and diastolic blood pressure, particularly in hypertensive subjects [32]. Another meta-analysis of 19 trials found that cocoa flavanols intake increased HDL cholesterol by 0.06 mmol/L and decreased C-reactive protein by 0.83 mg/mL [6]. Randomized feeding trials have reported that intake of high-flavanol dark chocolate produced reductions in platelet aggregation as well as improvements in endothelial function [33], and this effect was mainly mediated by a metabolite of flavanol (i.e., epicatechin) [34]. Other bioactives in chocolate—particularly methylxanthines—have also been shown to enhance the effects of cocoa flavanols on cardiovascular function [35]. In addition, flavanols in chocolate have been reported to have the ability of improving insulin sensitivity [6], possibility by promoting the survival and function of pancreatic β-cells and improving the insulin signaling pathway in hepatic cells [36,37]. Therefore, the aforementioned factors in combination may account for the reduced risks of CHD, stroke, and diabetes in our study.

In dose–response analysis, we detected a J-shaped relationship between chocolate intake and incident diabetes. Consuming chocolate in moderation (1–6 servings/week) is associated with a decreased risk of diabetes, while a higher than moderate intake cannot provide such a benefit. This dose–response pattern was also identified in a recent meta-analysis, which showed that light-to-moderate—but not high—consumption of chocolate has a protective role against heart failure, an end stage of cardiac disease [38]. For CHD and stroke, although the dose–response pattern was not J-shaped, there appeared little further risk reduction with moderate-to-high intake of chocolate (≥3 servings/week). All these features pointed towards the necessary cautions about the adverse effects of high or excessive chocolate intake. When consuming chocolate products in relatively high amounts, the high calorie content of chocolate will probably cause weight gain—an established risk factor for hypertension, diabetes, dyslipidemia, and cardiovascular disease [31]. Furthermore, higher consumption of chocolate may lead to less energy intake from other dietary foods that are helpful for the prevention of cardiometabolic disorders. In summary, the benefits of flavanols seem to be attenuated or even outweighed by the unfavorable effects of high energy intake when consuming high or excessive commercially available chocolate products.

Some limitations should be acknowledged. First of all, recall and selection biases may be present in our study due to the observational nature of individual studies. Nevertheless, we included only studies with a prospective design and pooled the maximally-adjusted risk estimates, which greatly reduced the probability of these biases. Second, due to the lack of data, we cannot conduct stratified analyses by some important confounders, such as total energy intake, body mass index, and types of chocolate products (milk, dark, or white). Third, dietary habits may also affect our final results. Subjects eating less chocolate are likely to have healthier dietary habits than those consuming chocolate in higher amounts. However, only few dietary factors were controlled for in the multivariate models of the original studies. Fourth, generalization of our findings is hampered by the geographical origin of the included studies, as they were mainly conducted in Europe and the United States.

## 5. Conclusions

Taken together, the present meta-analysis suggests that chocolate consumption confers reduced risks of CHD, stroke, and diabetes. Consuming chocolate in moderation (1–6 servings/week) may be optimal for the prevention of these burdensome diseases. Additional large prospective studies are required to confirm the observed benefits of chocolate in populations with different characteristics and to establish the optimum frequency of chocolate intake for preventing cardiometabolic disease.

## Figures and Tables

**Table 1:** Baseline characteristics of included studies.

Study	Population	*N*	Age	Ascertainments		Country	FU (Years)	Included Outcomes	Adjusted Factors
				Exposure	Outcome				
Buijsse 2010 [16]	General population	19,357	35–65	FFQ	ICD-10 code	Germany	8.1	CHD and stroke	Age, sex, smoking, drinking, BMI, diabetes, waist circumstance, employment status, physical activity, education, dietary energy, and food groups
Crichton 2017 [17]	Subjects without psychiatric illness and alcoholism	590	62 (mean)	FFQ	Standard assay methods	US	4.7	Diabetes	Age, sex, race, education, BMI, cholesterol level, hypertension, C-reactive protein, physical activity, grains, coffee, and red wine
Dong 2017 [18]	Subjects without CVD, diabetes, and cancer	84,597	44–76	FFQ	Predefined diagnostic criteria	Japan	12.9	Stroke	Age, area, BMI, dietary energy, smoking, drinking, sports, occupation, medication use, and food groups
Greenberg 2015 [19]	Community-based adults	7802	45–64	FFQ	Medical records of diabetic medication	US	13.3	Diabetes	Age, sex, race, smoking, drinking, physical activity, dietary energy, Keys Index of Dietary Quality, family history of diabetes, and educational and occupational levels
Greenberg 2017 [20]	Postmenopausal women	92,678	50–79	FFQ	Self-report of diabetic medication usage	US	13.1	Diabetes	Age, race, WHI Studyarm, physical activity, smoking, family history of diabetes, coffee, non-chocolate energy intake, Alternative Modified Health Eating Index, education, family income, and physical functional ability
Janszky 2009 [21]	Non-diabetic patients with post MI	1169	45–70	Self-report	ICD-9 and 10 codes	Sweden	8.7	CHD and stroke	Age, sex, smoking, drinking, BMI, physical activity, coffee intake, education, and sweet score
Kwok 2015 [22]	General population	20,951	59 (mean)	FFQ	ICD-10 code	UK	11.9	CHD and stroke	Age, sex, smoking, drinking, physical activity, dietary energy, diabetes, BMI, systolic BP, and cholesterol level
Larsson 2011 [23]	Women with no history of CVDs, diabetes, and cancer	33,372	49–83	FFQ	ICD-10 code	Sweden	10.4	Stroke	Age, smoking, drinking, BMI, education, physical activity, aspirin use, dietary energy, food groups, and history of hypertension, MI, and AF
Larsson 2012 [24]	General male population	37,103	45–79	FFQ	ICD-10 code	Sweden	10.2	Stroke	Age, smoking, drinking, BMI, education, physical activity, aspirin use, dietary energy, food groups, and history of hypertension, MI, and AF
Larsson 2016 [13]	Subjects without CVDs	67,640	45–83	FFQ	ICD-10 code	Sweden	13	CHD	Age, smoking, drinking, BMI, education, physical activity, exercise, aspirin use, dietary energy, food groups, and history of hypertension, MI, and AF
Lewis 2010 [25]	Older women	1216	NA	FFQ	ICD-10 code	Australia	9.5	CHD	Age, socioeconomic status, dietary energy, and BMI
Matsumoto 2015 [26]	Male physicians	18,235	40–84	FFQ	Self-report validated by medical records	US	9.2	Diabetes	Age, cohort status, smoking, drinking, exercise, BMI, dietary energy, and food groups
Mink 2007 [27]	Women without heart disease	34,489	55–69	FFQ	ICD-9 code	US	16	CHD and stroke	Age, smoking, dietary energy, marital status, education, BP, diabetes, BMI, waist-to-hip ratio, physical activity, and estrogen use
Oba 2010 [28]	Men and women without CVDs and cancer	13,540	≤70	FFQ	Self-reports	Japan	10	Diabetes	Age, smoking, drinking, dietary energy, BMI, physical activity, education, fat intake, women’s menopausal status

AF, atrial fibrillation; BMI, body mass index; BP, blood pressure; CHD, coronary heart disease; CVDs, cardiovascular diseases; FFQ, food-frequency questionnaire; FU, follow-up; ICD, International Classification of Disease; MI, myocardial infarction; NA, not applicable; WHI, Women’s Health Initiative.
